# *Apolipoprotein-*ε*4* is associated with higher fecundity in a natural fertility population

**DOI:** 10.1126/sciadv.ade9797

**Published:** 2023-08-09

**Authors:** Benjamin C. Trumble, Mia Charifson, Tom Kraft, Angela R. Garcia, Daniel K. Cummings, Paul Hooper, Amanda J. Lea, Daniel Eid Rodriguez, Stephanie V. Koebele, Kenneth Buetow, Bret Beheim, Riana Minocher, Maguin Gutierrez, Gregory S. Thomas, Margaret Gatz, Jonathan Stieglitz, Caleb E. Finch, Hillard Kaplan, Michael Gurven

**Affiliations:** ^1^ School of Human Evolution and Social Change, Arizona State University, Tempe, AZ, USA.; ^2^ Center for Evolution and Medicine, Arizona State University, Tempe, AZ, USA.; ^3^ Department of Population Health, New York University Grossman School of Medicine, New York City, NY, USA.; ^4^ Anthropology Department, University of Utah, Salt Lake City, UT, USA.; ^5^ Scientific Research Core, Phoenix Children's Hospital, Phoenix, AZ, USA.; ^6^ Department of Child Health, University of Arizona, Tucson, AZ, USA.; ^7^ Department of Health Economics and Anthropology, Economic Science Institute, Argyros School of Business and Economics, Chapman University, Orange, CA, USA.; ^8^ Child and Brain Development Program, Canadian Institute for Advanced Research, Toronto, Ontario, Canada.; ^9^ Department of Biological Sciences, Vanderbilt University, Nashville, TN, USA.; ^10^ Universidad de San Simon, Cochabamba, Bolivia.; ^11^ School of Life Sciences, Arizona State University, Tempe, AZ, USA.; ^12^ Max Planck Institute for Evolutionary Anthropology, Leipzig, Germany.; ^13^ Tsimane Gran Consejo, San Borja, Bolivia.; ^14^ MemorialCare Health System, Fountain Valley, CA, USA.; ^15^ University of California, Irvine, CA, USA.; ^16^ Center for Economic and Social Research, University of Southern California, Los Angeles, CA, USA.; ^17^ Institute for Advanced Study in Toulouse, Université Toulouse 1 Capitole, Toulouse, France.; ^18^ Leonard Davis School of Gerontology and Dornsife College, University of Southern California, Los Angeles, CA, USA.; ^19^ Department of Anthropology, University of California Santa Barbara, Santa Barbara, CA, USA.

## Abstract

In many populations, the a*polipoprotein-*ε*4* (*APOE-*ε*4*) allele increases the risk for several chronic diseases of aging, including dementia and cardiovascular disease; despite these harmful effects at later ages, the *APOE-*ε*4* allele remains prevalent. We assess the impact of *APOE-*ε*4* on fertility and its proximate determinants (age at first reproduction, interbirth interval) among the Tsimane, a natural fertility population of forager-horticulturalists. Among 795 women aged 13 to 90 (20% *APOE-*ε*4* carriers), those with at least one *APOE-*ε*4* allele had 0.3 to 0.5 more children than (ε3/ε3) homozygotes, while those with two *APOE-*ε*4* alleles gave birth to 1.4 to 2.1 more children. *APOE-*ε*4* carriers achieve higher fertility by beginning reproduction 0.8 years earlier and having a 0.23-year shorter interbirth interval. Our findings add to a growing body of literature suggesting a need for studies of populations living in ancestrally relevant environments to assess how alleles that are deleterious in sedentary urban environments may have been maintained by selection throughout human evolutionary history.

## Teaser

Among natural fertility forager-farmers, women with the *APOE-*ɛ*4* allele have more children, and the effect is dose dependent.

## INTRODUCTION

In European populations, the apolipoprotein-ε4 (*APOE-*ε*4*) allele is associated with increased incidence and mortality from several deleterious health conditions, including Alzheimer’s type dementia, and cardiovascular disease (*1*–*5*). Despite these negative health consequences in many, but not all populations (*6*), the *APOE-*ε*4* allele, which is ancestral in humans, has a prevalence ranging between 5 and 45% across populations (*7*). Its persistence over many generations, despite well-documented harms, is an evolutionary puzzle. One reasonable solution to the puzzle would be that fertility benefits might offset any costs to (late life) survival. To date, however, there has been a paucity of research on the reproductive effects of the apolipoprotein isoforms, especially in natural fertility settings (see Table 1 for a summary).

**Table 1.** A summary of previous studies on E4-fertility relationships in humans. The two rows in bold indicate the present study. N/A, not available.

Study	High-pathogen exposure	*N*	Fertility (# births)				Controlled fertility	Notes
			No *APOE-*ε*4*	One *APOE-*ε*4*	*APOE-*ε*4*/*APOE-*ε*4*	Any *APOE-*ε*4* vs. No *APOE-*ε*4*		
Ghana (low pathogen) (*20*)	N	197	7.52	7.42	6.39	−0.2 fewer children	Low	Fertility measured in women over age 40
Italy (*32*)	N	160	3.73	3.70	N/A	−0.03 fewer children	Yes	Postmenopausal cohort
Poland (*34*)	N	118	1.01	N/A	N/A	−0.33 fewer children	Yes	Young cohort mean age 30 years
Denmark (*33*)	N	370	1.88	N/A	1.5	−0.38 fewer children	Yes	Male cohort—all other studies female
Ghana (high pathogen) (*63*)	Y	40	7.53	8.35	11.95	1.15 more children	Low	Fertility measured in women over age 40
Afro-Ecuadorians (*21*)	Y	57	6.79	8.64	6.7	1.42 more children	Natural fertility	Mean age 39
African-Cayapa (*21*)	Y	27	6.3	6.2	6.3	−0.03 fewer children	Natural fertility	Mean age 39
**Tsimane (all ages)**	**Y**	**795**	**7.83**	**7.99**	**9**	**0.23 more children**	**Very low**	**All age cohort (median age 47)**
**Tsimane (completed fertility)**	**Y**	**355**	**9.46**	**9.90**	**11.33**	**0.53 more children**	**Natural fertility**	**Completed fertility cohort**

One possibility for the maintenance of *APOE-*ε*4* is that its harmful cardiovascular and neurodegenerative effects are largely restricted to older, post-reproductive ages in the “selection shadow” (*8*–*10*). Such an explanation is consistent with mutation accumulation theory, which underlies much of the literature on the evolution of senescence (*11*, *12*). Applied to the *APOE* gene, mutation accumulation assumes no major fitness effects of *APOE-*ε*4* versus of *APOE-*ε*3* before reproductive cessation, with most of the cardiovascular and cognitive effects occurring at late ages. An alternative view, relying on antagonistic pleiotropy, hypothesizes that the possible advantages of *APOE-*ε*4* in high-pathogen settings may be in balance with later life costs (*13*, *14*). In particular, in high-pathogen settings, *APOE-*ε*4* has favorable impacts on immune function and growth (*15*, *16*), cognition (*17*–*19*), and fertility (*20*–*22*) (Table 1). Demonstration of fitness benefits, especially those in early life, are consistent with *APOE-*ε*4* persistence maintained by antagonistic pleiotropy (*13*).

Previous studies have suggested several mechanisms by which *APOE-*ε*4* could impact survival and fertility, including faster growth and development (and thus earlier age at first reproduction), greater adiposity, and improved immune function. *APOE-*ε*4* was initially recognized clinically in association with elevated cholesterol and cardiovascular disease (*23*). While higher lipids may increase cardiovascular disease risk, they also may be protective against some pathogens (*24*). For example, *APOE-*ε*4* is associated with resistance to giardia (*15*, *18*), spontaneous clearance of cryptosporidium (*16*), and resistance to hepatitis C (*25*) and plays other important immunomodulatory roles (*26*). A higher pathogen burden early in life is linked to slower growth rates, worse nutritional outcomes, and later reproduction (*27*). Children who are *APOE-*ε*4* carriers living in low-income settlements in Brazil with high infection rates grow faster than non–*APOE-*ε*4* carrier children (*15*, *18*, *28*). Overall, *APOE-*ε*4* appears to be beneficial to phenotypic condition in several populations facing high parasite and pathogen loads; improved phenotypic condition could also have downstream benefits for earlier or more frequent reproduction. These potential benefits of the *APOE-*ε*4* allele would not be evident in urban industrialized environments with low parasite or pathogen loads. In those contexts, only the negative impacts of the *APOE-*ε*4* allele would be observed.

In natural fertility populations, higher fertility can be achieved several ways, often referred to as “proximate determinants”: earlier sexual maturity and age at first reproduction, shorter interbirth intervals (IBIs), and later age at last reproduction (*29*, *30*). Mothers with higher adiposity or those with access to complementary feeding can have shorter IBIs, while IBIs are increased in the case of fetal loss (*31*). The use of contraceptives affects fertility for reasons unrelated to fecundity such that settings characterized by controlled fertility are unlikely to reveal fertility benefits of *APOE-*ε*4*. Several studies of urban, low-fertility populations showed no positive effect of *APOE-*ε*4* alleles on fertility (*20*, *32*–*34*). However, the few studies conducted in natural fertility populations or high-fertility populations support fertility-related benefits of the *APOE-*ε*4* allele (*20*, *21*, *34*), although inferences to date are limited by small sample size, a lack of attention to the proximate determinants of fertility, and limited assessment of *APOE-*ε*4* in high-pathogen environments (Table 1).

Here, we test the functional role of the *APOE-*ε*4* allele on several measures of reproductive success in a natural fertility population, the Tsimane of Bolivia. Tsimane forager-horticulturalists are an ideal population to test *APOE-*ε*4*–fertility relationships, given their active subsistence lifestyle, high natural fertility and intensive on-demand breastfeeding, historical lack of access to effective birth control, and a relatively large sample size compared to previous studies. In addition to total fertility, our attention to ages of menarche, first birth, and interbirth intervals may provide insight into possible mechanisms underlying proposed *APOE-*ε*4*–fertility relationships.

## RESULTS

Our sample includes 795 Tsimane women aged 13 to 90 (median age, 47.3 years old; SD, 15.8) (Table 2). Of these women, 80.0% are homozygous for the *APOE-*ε3 allele (ε3/ε3) and 18.5% heterozygous (ε3/ε4) and 1.5% homozygous for the *APOE-*ε*4* allele (ε4/ε4). The *APOE-*ε*2* allele is not detected among the Tsimane population (*35*). In total, 20.0% of participants had at least one copy of *APOE-*ε*4*. The median fertility in this sample was 9.0 live births per woman (SD, 4.3); for women over the age of 45 with completed fertility, the median total fertility was 10.0 live births per woman (SD, 3.5).

**Table 2.** Participant characteristics for the current study.

	Full sample			*APOE-*ε*3/APOE-*ε*3*		*APOE-*ε*3/APOE-*ε*4*		*APOE-*ε*4/APOE-*ε*4*	
	*n*	Median	SD	Median	SD	Median	SD	Median	SD
Fertility	795	9.0	4.3	8.0	4.2	9.0	4.4	10.5	4.0
Age (years)	795	47.3	15.8	47.5	15.7	46.4	15.9	43.7	17.2
Age at first reproduction	392	17.9	2.9	18.0	3.0	17.3	2.7	14.6	3.2
Average interbirth interval	355	2.2	0.7	2.2	0.7	2.0	0.5	2.2	0.0
Age at last reproduction	505	40.4	4.5	40.5	4.6	40.0	4.4	41.4	5.5
Age of menopause	235	49.0	4.5	50.0	4.5	48.0	4.2	50.0	3.2
Fetal loss	734	0.0	1.3	1.0	1.3	0.0	1.1	0.0	1.4
Age of menarche	680	13.0	0.9	13.0	0.9	13.0	1.0	14.0	1.0
Height (cm)	795	150.0	4.8	150.0	4.9	150.6	4.7	150.1	6.4
Weight (kg)	795	53.3	10.6	52.9	10.4	55.5	10.9	53.5	10.4
BMI (kg/m^2^)	795	23.3	4.1	23.2	4.0	24.2	4.3	23.4	3.9

In addition to total fertility, data on age at first reproduction, completed fertility, and age at last reproduction from detailed demographic interviews allowed calculation of the average IBI for a subset of Tsimane women with completed fertility (*n* = 355; Table 2). Anthropometric data were collected as a proxy for maternal condition. Because fertility is asymptotic (e.g., does not increase monotonically with age post-reproductively), 1/age was used for age adjustment. We used two types of statistical approaches: frequentist and Bayesian. Frequentist regression models with Poisson distributions were used to examine associations between *APOE* genotypes and fertility. *APOE* genotypes were modeled both as a binary (no *APOE-*ε*4* versus at least one *APOE-*ε*4* allele) and as total *APOE-*ε*4* copies (0, 1, 2). A second confirmatory analysis was conducted with Bayesian modeling. This latter approach uses a Gaussian process to model the probability of birth over the life course, to detect the timing of fertility effects of *APOE-*ε*4*. The results were consistent between modeling approaches.

### *APOE-*ε*4 and phenotypic condition*

Women with at least one copy of the *APOE-*ε*4* allele trended toward being 1.8 kg heavier than women without the *APOE-*ε*4* allele [95% confidence interval (CI), 0.0 to 3.6; *P* = 0.054], controlling for age. *APOE-*ε*4* carriers were also more likely to be overweight or obese [body mass index (BMI) ≥ 25 kg/m^2^] [odds ratio (OR), 1.6; *P* = 0.015; 95% CI, 1.1 to 2.2], controlling for age.

### *APOE-*ε*4 and fertility*

**Fig. 1.**
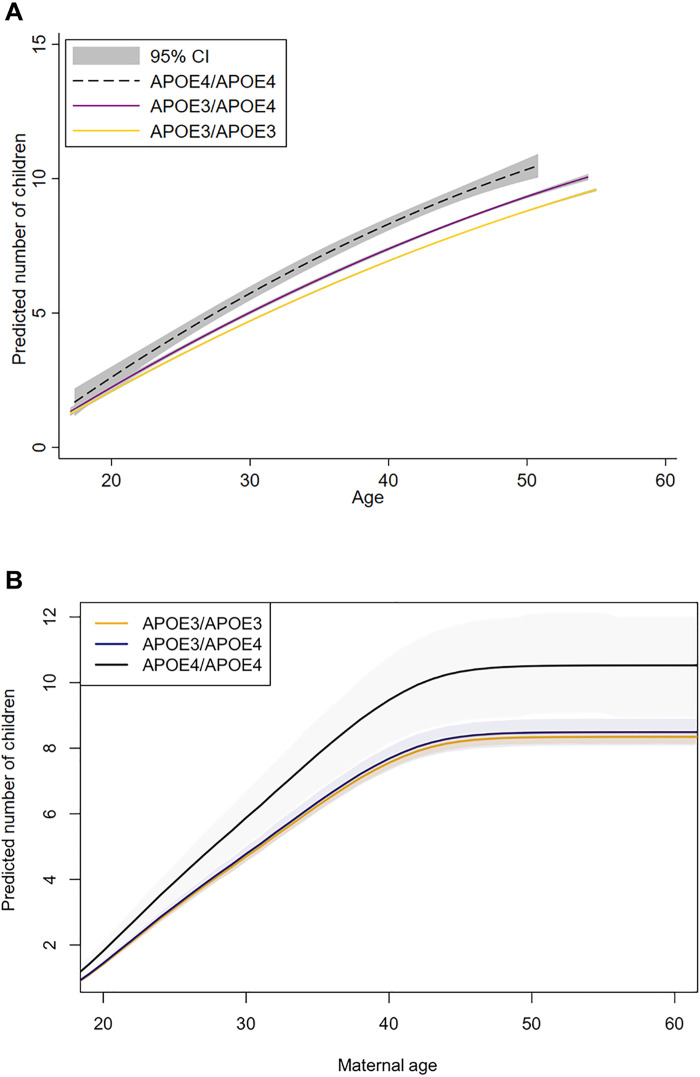
Associations between *APOE* genotype and fertility in 795 Tsimane women aged 13 to 90. (**A**) Based on Poisson regressions. (**B**) Based on Bayesian linear regression.

Controlling for 1/age and BMI, Tsimane women with at least one copy of the *APOE-*ε*4* allele had 0.5 more children at the median age of 47.3 than those who were homozygous for *APOE-*ε3 (*P* = 0.033; 95% CI, 0.3 to 0.7; table S1). When heterozygous (ε3/ε4) and homozygous (ε4/ε4) *APOE-*ε*4* genotypes were analyzed separately, individuals with one copy of *APOE-*ε*4* trended toward 0.4 more children (*P* = 0.084; 95% CI, 0.2 to 0.6; table S2), and those with two copies trended toward 1.4 more children (table S1; *P* = 0.053; 95% CI, 0.1 to 2.8) compared to *APOE-*ε3 homozygotes (Fig. 1A).

Similarly, confirmatory Bayesian models found that Tsimane woman of average BMI with at least one copy of the *APOE-*ε*4* allele had approximately 0.3 more children than women homozygous for the *APOE-*ε3 allele, by age 47.3 years (95% CI, −0.18 to 76). Comparing women of each genotype, individuals with two copies of the *APOE-*ε*4* allele had 2.2 more children (95% CI, 0.66 to 3.71) than those without an *APOE-*ε*4* allele, while women with one copy had 0.14 more children (95% CI, −0.30 to 0.60) than those without an *APOE-*ε*4* allele. While these effects on cumulative births become evident by the second to third decade of life (Fig. 1B), differences in the annual probability of birth are evident even earlier (fig. S2).

Women in this sample self-reported between 0 and 10 miscarriages or stillbirths (mean, 0.9; 95% CI, 0.8 to 1.0) (see the Supplementary Materials for further details). Controlling for 1/age and BMI, women with at least one copy of the *APOE-*ε*4* allele were less likely to experience self-reported fetal loss (OR, 0.6; *P* = 0.013; 95% CI, 0.4 to 0.9; fig. S3). Zero-inflated Poisson models controlling for 1/age and BMI show that *APOE-*ε*4* is associated with a lower probability of self-reported fetal loss [incidence rate ratio (IRR), 0.7; *P* = 0.009; 95% CI, 0.2 to 1.2; fig. S3], but not with the number of fetal losses for those who experienced loss. The probability of self-reported fetal loss increases with age among the Tsimane (*36*), and so additional analyses were conducted for women with completed fertility. Consistent with the analysis focusing on the fuller sample, post-reproductive age women with the *APOE-*ε*4* allele were less likely to have reported fetal loss earlier in life (OR, 0.5; *P* = 0.010; 95% CI, 0.3 to 0.9; IRR, 0.8; *P* = 0.008; 95% CI, 0.2 to 1.4).

### *APOE-*ε*4* and proximate determinants of fertility

**Fig. 2.**
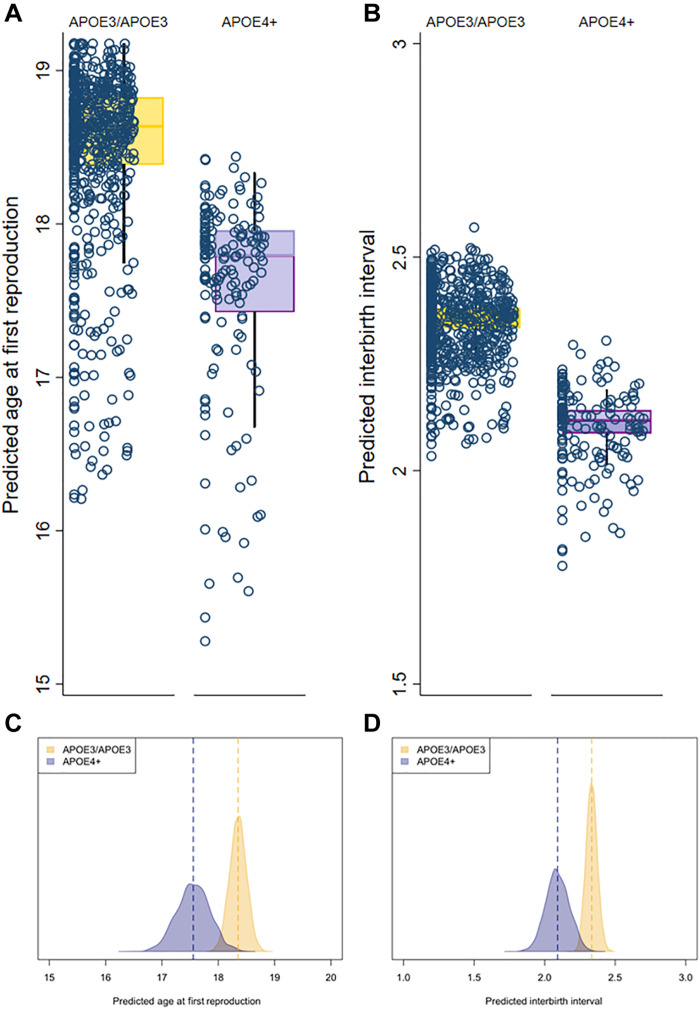
Variation in the proximate determinants of fertility, including age at first reproduction and interbirth interval by *APOE* genotype. The top panel uses frequentist statistics for age at first reproduction (**A**) and interbirth interval (**B**), while the bottom panel represents the Bayesian results for age at first reproduction (**C**) and interbirth interval (**D**).

Women with at least one *APOE-*ε*4* allele had a 10.3% shorter IBI (mean, 0.24 years; *P* = 0.013; 95% CI, 0.05 to 0.4 years shorter), controlling for 1/age and BMI (Fig. 2B). When heterozygous (ε3/ε4) and homozygous (ε4/ε4) *APOE-*ε*4* carriers were analyzed separately, having one copy of the *APOE-*ε*4* allele was associated with a 10.4% reduction in IBI (mean, 0.24 years shorter; *P* = 0.014; 95% CI, 0.0 to 0.4 years shorter), while having two copies of the *APOE-*ε*4* allele was associated with a 6.9% reduction in IBI (mean, 0.2 years shorter; *P* = 0.741; 95% CI, 1.1 to 0.8 years longer).

Bayesian models produced similar results for IBI. Women with at least one copy of the *APOE-*ε*4* allele had a 10.3% shorter IBI (0.24 years shorter; 95% CI, 0.05 to 0.42 years), controlling for BMI. Having one copy of the *APOE-*ε*4* allele was associated with a reduction in IBI of 0.23 years (95% CI, 0.05 to 0.41 years) compared with ε3/ε3 women, while having two copies of the *APOE-*ε*4* allele was associated with a reduction in IBI of 0.46 years (95% CI, −0.28 to 1.2 years).

Age at first reproduction was 0.8 years earlier (*P* = 0.029; 95% CI, 0.1 to 1.5 years earlier; Fig. 2C) for women with at least one copy of the *APOE-*ε*4* allele, controlling for 1/age and BMI. When heterozygous (ε3/ε4) and homozygous (ε4/ε4) *APOE-*ε*4* carriers were analyzed separately, one copy of the *APOE-*ε*4* allele was associated with a 0.7-year earlier age at first reproduction (*P* = 0.059; 95% CI, 0.0 to 1.5 years earlier), while two copies were associated with a 2.1-year earlier reproductive debut (*P* = 0.100; 95% CI, 4.7 years earlier to 0.4 years later). There was no association between *APOE-*ε*4* status and age at last reproduction (*P* = 0.924).

Bayesian models also confirm an earlier age at first reproduction for women with at least one copy of the *APOE-*ε*4* allele, approximately 0.79 years earlier (95% CI, 0.17 to 1.45 years earlier), controlling for BMI. Comparing homozygous (ε4/ε4) and heterozygous (ε3/ε4) carriers of the *APOE-*ε*4* allele with *APOE-*ε3 homozygotes, one copy of the allele was associated with an age at first reproduction 0.65 years earlier (95% CI, −0.04 to 1.29 years earlier) and two copies of the allele were associated with an age of reproduction 4.0 years earlier (95% CI, 2.40 to 5.62 years earlier).

### *APOE-*ε*4, menarche, and reproductive span*

While women with at least one copy of the *APOE-*ε*4* allele had an earlier age at first reproduction, there was no association between *APOE-*ε*4* status and self-reported age at menarche (*P* = 0.292), controlling for age and BMI. Age at menarche (*P* = 0.221) was not associated with total fertility. Reproductive span was calculated by subtracting age at last reproduction from age at first reproduction, and was not associated with *APOE-*ε*4*.

## DISCUSSION

In this relatively large sample of women living in a subsistence environment, women with at least one copy of *APOE-*ε*4* exhibited higher fertility than *APOE-*ε3 homozygotes. On average, Tsimane women with at least one *APOE-*ε*4* allele had 0.3 to 0.5 more children than ε3/ε3 carriers, while those with two *APOE-*ε*4* alleles gave birth to 1.4 to 2.1 more children than ε3/ε3 carriers. *APOE-*ε*4* was associated with earlier reproduction and having shorter IBIs than women who are homozygous for *APOE-*ε3. Not only did carriers of *APOE-*ε*4* begin reproducing earlier, but they also weighed more and had greater BMI, consistent with both increased maternal condition affecting fertility. Previous studies have shown a positive association between *APOE-*ε*4* and growth in children (*15*). While higher BMI is generally considered unhealthy at the higher range in industrialized populations, higher BMI may be advantageous for supporting reproduction in subsistence populations like the Tsimane with minimal obesity.

The sizable and dose-dependent effects of the presence of *APOE-*ε*4* on Tsimane fertility are similar, though slightly smaller than those reported in rural Ghana (*20*). In that study, Ghanaian women with one copy of *APOE-*ε*4* bore an additional 1.02 children, while those with two copies had 3.47 more children than noncarriers (*20*). Similarly, a separate study with a small number of Afro-Ecuadorian women (*n* = 57, 27 with an *APOE-*ε*4* allele) and Cayapa women (*n* = 27, 14 with an *APOE-*ε*4* allele) also found that women with one copy of the *APOE-*ε*4* allele had an age-adjusted 2.2 more children than homozygous *APOE-*ε3 women (*21*) (Table 1). While our results show slightly smaller effects of *APOE-*ε*4* on fertility, the sample size in previous studies in high-pathogen environments were relatively small (ranging from 27 to 57 individuals). These populations vary in age, marital practices, and parasite and pathogen load, and so we are unable to separate causal differences due to these factors. Thus, the studies to date across multiple low-contracepting and high-pathogen populations all show similar effects, suggesting that in high-pathogen natural fertility contexts, the *APOE-*ε*4* allele may lead to higher fertility. While the sample sizes for homozygotic *APOE-*ε*4* individuals are small in all three studies, two of three studies report gene dose-dependent effects. In contrast, the fertility effects of the *APOE-*ε*4* allele appear to be diminished in low pathogen settings, particularly those with access to contraceptives in urban environments (*20*, *21*, *32*–*34*) (Table 1).

In natural fertility populations, two of the critical proximate determinants of lifetime fertility are age at first birth and IBIs. The length of the IBI is based on a mix of factors, including fecund waiting time to conception, lactational amenorrhea, and recovery time following fetal loss (*30*). Among the Tsimane, breastfeeding is ubiquitous, but complementary foods are often introduced at a relatively early age (~4 months), with weaning complete by 27 months (*37*). Here, we found that Tsimane women with at least one *APOE-*ε*4* allele start reproducing at earlier ages, in more rapid succession. While *APOE-*ε*4* is generally thought to be detrimental in urban European contexts due to high blood lipid levels, increased cardiovascular disease risk, and higher rates of dementia (*26*, *38*, *39*), the Tsimane generally have low levels of lipids, cardiovascular disease, and dementia (*40*, *41*).

*APOE-*ε*4* carriers may be better able to clear infections in both humans and experimental rodent models (*15*, *16*, *28*, *42*). Minimizing parasites and infectious pathogens reduces energetic constraints for child growth and thus earlier age at first reproduction, and could lead to positive energy balance, which is critical for resuming ovulation following lactational amenorrhea (*43*). Normally, earlier age at first birth should trade off against adult height; energy spent on growth cannot be spent on reproduction, and vice versa. However, potentially lower parasite load and higher BMI in *APOE-*ε*4* carriers may ease energetic constraints, allowing women to grow rapidly, and to give birth at an earlier age, and at a faster rate, than their noncarrier peers. While the median BMI in these women is 23.3 kg/m^2^, it should be noted that BMI is overestimated for short-statured, lean individuals (*44*). There has been increasing access to market goods over the last two decades (especially sugar and cooking oil); the use of these is low and was likely very low for post-reproductive women; for example, in 2010, only 26.2% of families reported buying cooking oil in the last month (*45*).

While there was no impact of *APOE-*ε*4* status on reported age at menarche, it should be noted that there is a period of adolescent subfecundity following menarche (*43*); thus, the reproductive benefits of *APOE-*ε*4* may only be fully realized once the reproductive system is matured, and the individual has entered into a reproductive union. It is also possible that middle-aged and post-reproductive women may not accurately remember the timing of menarche. Similarly, the self-reported age at last menses could also be less accurate, particularly given that the transition to menopause is a multiyear process (*46*). Further, there is no cultural discourse around menopause, nor a Tsimane word for menopause, which makes self-reported age at the final menstrual period extremely difficult to assess, and most women were not able to self-report their exact age at menopause. As such, we could not assess associations between *APOE-*ε*4* and age at menopause.

Natural selection works on thin margins―a single allele resulting in a large increase in fertility would quickly reach fixation, yet in this population the *APOE-*ε*4* allele appears to be in Hardy-Weinberg equilibrium (table S3), with about 20% of individuals carrying the allele and 1.5% being homozygous. This raises the question of what kinds of balancing forces may be responsible for this frequency. First, these fertility effects may be most evident during periods of rapid population growth, much like the Tsimane are currently experiencing. Throughout most of human history as hunter-gatherers, there was relatively low population growth due to high mortality, periodic catastrophes, and energy limitations affecting fertility (*47*); with small-scale horticulture, including rice introduced by the Jesuits, the Tsimane have access to high-quality weaning foods, increasing market access, and face relatively low (compared to the human past) mortality. The Tsimane also experience higher parasite and pathogen loads then either urban industrial settings or hunter-gatherer populations, and thus, the fertility effects may be more evident in this population due to more extreme immunological stressors (*48*–*50*). Thus, it may be that the *APOE-*ε*4* allele would have had much less of an impact on reproductive success when total fertility was low, but with a total fertility rate of ~9 among the Tsimane, the effect on fertility becomes substantial. A second possibility is that there are pleiotropic effects of the *APOE-*ε*4* allele, resulting in higher rates of early life mortality. Similar to Mostafavi *et al.* (*51*), we find that a smaller proportion of Tsimane women have the *APOE-*ε*4* allele at later ages (see fig. S4). Another potential possibility would be to examine hypothesized negative effects on male fertility (*52*). Finally, the negative impacts of the *APOE-*ε*4* allele at older ages may have played an important role in its selection. We have documented that older age individuals play an important role in subsidizing the food supply of children and grandchildren, both among the Tsimane and other subsistence populations (*53*, *54*). This raises the intriguing possibility that the evolution of the *APOE-*ε3 allele may be related to brain expansion and the maintenance of cognitive abilities and subsistence productivity in old age. These possibilities are not mutually exclusive and will be examined in future studies.

Few studies have addressed apolipoprotein isoforms and reproduction. While the *APOE-*ε*2* isoform has not been detected among the Tsimane (*35*), it is possible that the longer life span noted among *APOE-*ε*2* carriers in some populations (*55*) may contribute to reproductive success via indirect reproduction (inclusive fitness; e.g., providing resources to support the fertility of their children) at later ages. The lower risk of cognitive decline in *APOE-*ε*2* and *APOE-*ε*3* carriers suggests a testable hypothesis that older individuals could support the fertility of their descendants. Given that multigenerational resource flows are common in subsistence populations, increased investment in descendent kin due to later survival of *APOE-*ε*2* carriers (*53*, *54*) could potentially have offset the higher fertility of *APOE-*ε*4* carriers. Other populations living under high infectious load should be evaluated for direct association of *APOE-*ε*2* with fecundity. In any case, comprehensive evaluation of how antagonistic pleiotropy might affect *APOE* allelic distributions will require a greater understanding of fitness-relevant costs and benefits at different ages, and across different environments. It is also a possibility that balancing selection has maintained the *APOE-*ε*4* given potential immune or lipid benefits of the allele in a high-pathogen environment (*26*).

As the vast majority of human evolutionary history occurred in small-scale hunter-gatherer populations with relatively high fertility and mortality, there may be a mismatch between the costs and benefits of particular genetic profiles in ancestral environments versus food-rich, sedentary, post-industrialized populations. It is a core tenet of evolutionary medicine that these evolutionary mismatches contribute to the growing incidence of chronic diseases of aging seen today (*48*, *56*). While no extant subsistence population is a perfect exemplar of the human past, most biomedical research is conducted in urban environments that are highly divergent from most of human existence. Studies in industrial urban populations find largely detrimental effects of *APOE-*ε*4* on cardiovascular and neurodegenerative conditions in aged individuals. However, despite more rapid and severe neural degeneration with age, *APOE-*ε*4* carriers show little evidence of detrimental effects before old age (*57*, *58*), and thus, if women with the *APOE-*ε*4* are reproducing more at earlier ages, this could underscore the potential for *APOE-*ε*4* maintenance through antagonistic pleiotropy. The few studies conducted in high-pathogen natural fertility settings are concordant with the benefits of *APOE-*ε*4* on various systems, including faster growth, pathogen resistance, and higher reproductive success. Other studies in natural fertility populations of putatively “deleterious” alleles have also reported fertility benefits; for example, the *BRCA1/2* allele is associated with a similar effect size (approximately two additional children) on fertility in U.S. women pre-1930 (*59*). Overall, this growing body of literature suggests a need for targeted studies in populations living in higher fertility environments more similar to those in which humans evolved, to assess the biological fitness-relevant consequences of common “deleterious” alleles. Potential links between such alleles and fertility could explain why some alleles persist today despite negative health outcomes in industrial populations.

## METHODS

The Tsimane are a population of ~17,000 forager-horticulturalists living in over 90 villages throughout the Bolivian Amazon lowlands. The Tsimane Health and Life History Project (THLHP) has been collecting longitudinal demographic and biomedical data since 2002 (*60*). A mobile medical team with trained anthropologists conducts biomedical surveillance, including biomarker collection (*49*), and reproductive histories (*61*–*63*). At the time of study, all fertility data reflect natural fertility, i.e., breastfeeding on demand, absence of effective contraception, and no visible regulation of parity. A previous study also confirms no secular changes in age-specific fertility among the cohorts represented in the sample (*62*).

### Ethics

Informed consent was collected at three levels: by the individual, by the community, and by the Tsimane Gran Consejo (Tsimane governing body). All study protocols were approved by the Institutional Review Boards of the University of New Mexico (#07-157) and the University of California Santa Barbara (#3-21-0652).

### Sample

Our sample includes 795 Tsimane women aged 13 to 90 (median age, 47.3 years old; SD, 15.6) who had *APOE* genotyped and demographic data collection. See fig. S1 for STROBE diagram.

### Genotyping

Whole blood was frozen in liquid nitrogen before transfer on dry ice to the University of California Santa Barbara [see (*26*) for additional details]. DNA was extracted by using standard protocols. Determination of the *APOE-*ε*2/*ε*3/*ε*4* alleles in the Tsimane was derived from genotypes of two single-nucleotide polymorphisms: rs429358 and rs7412. Genotyping was performed using the TaqMan Allelic Discrimination system (Thermo Fisher Scientific, Carlsbad, CA, USA). Note that the *APOE-*ε*2* allele is not detected among the Tsimane population (*35*).

### Reproductive histories

During routine yearly health screening from 2002 to 2022, women are asked in the Tsimane language about the total number of live births they have ever had, live births since their last health screening, the total number of pregnancies, and pregnancies since their last screening. Women are also asked when they underwent menarche, and the date of their last menstrual cycle, which is used to back-calculate if that woman was menopausal if she had not had a menstrual cycle for 1 year (there is no word in the Tsimane language for menopause). Additionally, a subset of women (*n* = 679) had detailed reproductive histories first collected in 2002–2005, which have been updated over the last two decades during medical surveillance from 2002 to 2022 (*61*, *64*). A combination of methods developed in other populations (*65*, *66*) was used to assign ages to mothers and offspring, including written records, dated events, family age lists, photo comparisons of people with known ages, and triangulating ages with independent interviews of kin (*64*). The Tsimane have no taboos about talking about the dead or fetal loss (see the Supplementary Materials). That said, early fetal loss is likely to be under-detected and underreported, especially in the first trimester (see limitations in the Supplementary Materials) (*36*). Total number of births from annual screenings and from reproductive histories are highly correlated in post-reproductive women (*R*^2^ = 0.922; *P* < 0.0001), and a Bland-Altman plot shows no directional bias.

We excluded any observations of women whose first birth was reported to have occurred after age 30 years, since it is possible that they experienced extenuating reproductive circumstances (e.g., secondary sterility and infertility), or there may have been errors in age determination. Our inclusion criteria include 95% of all births according to other studies of Tsimane fertility (*61*–*63*).

Age at menarche was self-reported, and there was some level of missingness for women who did not remember age at menarche (14.6%). All anthropometric measurements (height, weight, and BMI) were collected at the same visit when reproductive questionnaires were performed, and a limitation of this study is the lack of mid-life anthropometrics for post-reproductive women.

### Anthropometrics

Height was collected by a physician using a SECA 213 portable stadiometer, and weight was captured with a Tanita BC-1500 scale.

### Statistical methods

All frequentist models were run in STATA 17.0, and all Bayesian models were fit using R (version 4.0.3) and the probabilistic programming language Stan (version 2.21.2). Poisson regression models examined associations between total fertility and *APOE* genotype, controlling for 1/age, and BMI. The 1/age variable was selected as it reaches an asymptote that better approximates reproductive cessation at menopause than linear age, log age, or quadratic age terms; it should be noted that results are robust to all age transformations. Linear regressions examined associations between *APOE* genotype and proximate determinants of fertility (IBI, age at first reproduction, age at last reproduction), as well as associations between *APOE* genotype and anthropometrics. It should be noted that the main results (fertility, age at first reproduction, and IBI) were robust regardless of which anthropometric control variables were included for maternal condition (height, weight, BMI). Exploratory associations between *APOE* genotype and fetal loss were examined both with a logistic regression (binary variable for any fetal loss) and with a zero-inflated Poisson model (see the Supplementary Materials).

A confirmatory Bayesian approach uses a Gaussian process to model the probability of birth over the life course. This approach also pools information about fertility probabilities with a Gaussian decay term by “distance” between ages, which provides a robustness check against age uncertainties and sample size differences. We assessed convergence using the R-hat convergence diagnostic, estimate of autocorrelation-adjusted number of samples, and visual inspections of the trace plots of chains for all parameters. We plot posterior predictions showing resulting 95% compatibility intervals.

## Supplementary Materials

### This PDF file includes:

Figs. S1 to S4 Tables S1 to S3 References
