# Supplementary material for: Apolipoprotein-ε4 is associated with higher fecundity in a natural fertility population
Source: Sci Adv. 2023 Aug 9;9(32):eade9797. doi: 10.1126/sciadv.ade9797 (PMC10411886; doi:10.1126/sciadv.ade9797)
Supplement: Supplementary file 1 — Figs. S1 to S4 Tables S1 to S3 References [file sciadv.ade9797_sm.pdf]

Science Advances
AAAS

Supplementary Materials for

Apolipoprotein-ε4 is associated with higher fecundity in a natural fertility population

Benjamin C. Trumble *et al.*

Corresponding author: Benjamin C. Trumble, btrumble@asu.edu; Jonathan Stieglitz, jonathan.stieglitz@iast.fr; Caleb E. Finch, cefinch@usc.edu; Hillard Kaplan, hkaplan@chapman.edu; Michael Gurven, gurven@anth.ucsb.edu

Sci. Adv. **9**, eade9797 (2023)
DOI: 10.1126/sciadv.ade9797

This PDF file includes:

Figs. S1 to S4
Tables S1 to S3
References

Figure S1: STROBE Diagram indicating sample data and sample sizes.

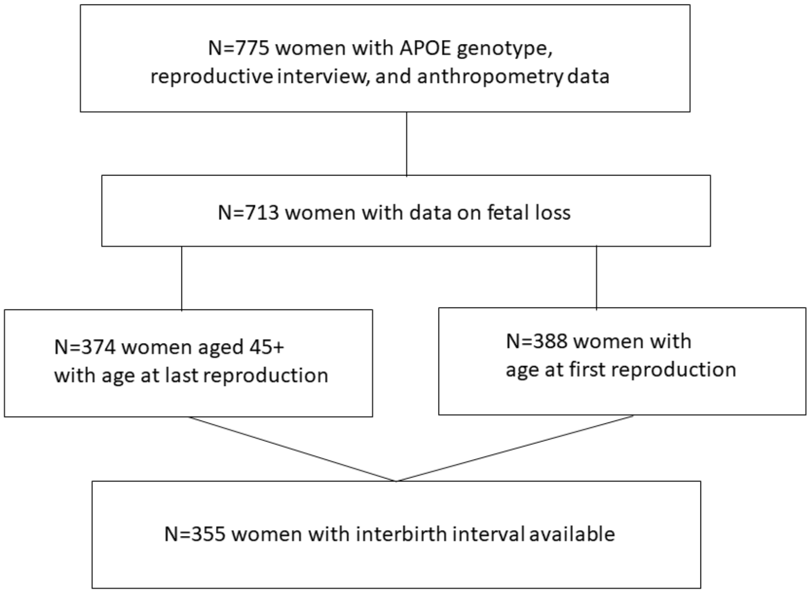

Figure S2: Predicted probability of a single birth event at a particular age, for women carrying two copies of the *APOE-ε4* allele (black), women carrying a single copy of the *APOE-ε4* allele (navy) and women who do not carry the allele (yellow). We observe a substantially increased probability of having a child for women with two copies of the allele, and an increased probability of having a child for women with one copy of the allele, compared with women who do not carry the allele, at all ages, controlling for BMI. The lines plot the posterior mean, and the shaded intervals plot the 95% compatibility region of the posterior interval. The empty circles plot the mean number of births recorded for women at a particular age, colored by the *APOE* genotype.

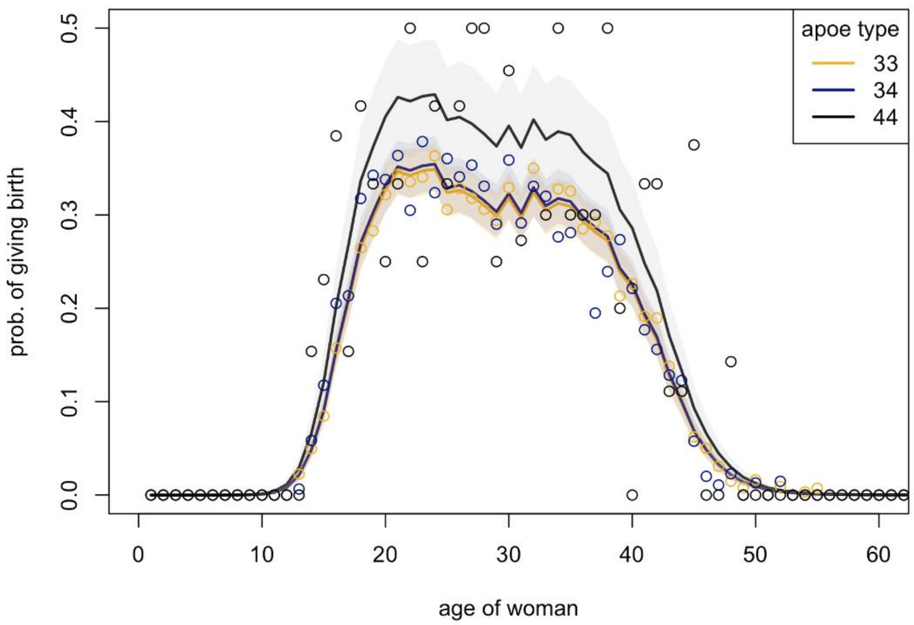

Figure S3: Predicted fetal loss by *APOE* genotype. The first panel uses frequentist statistics, while the second panel represents the Bayesian results

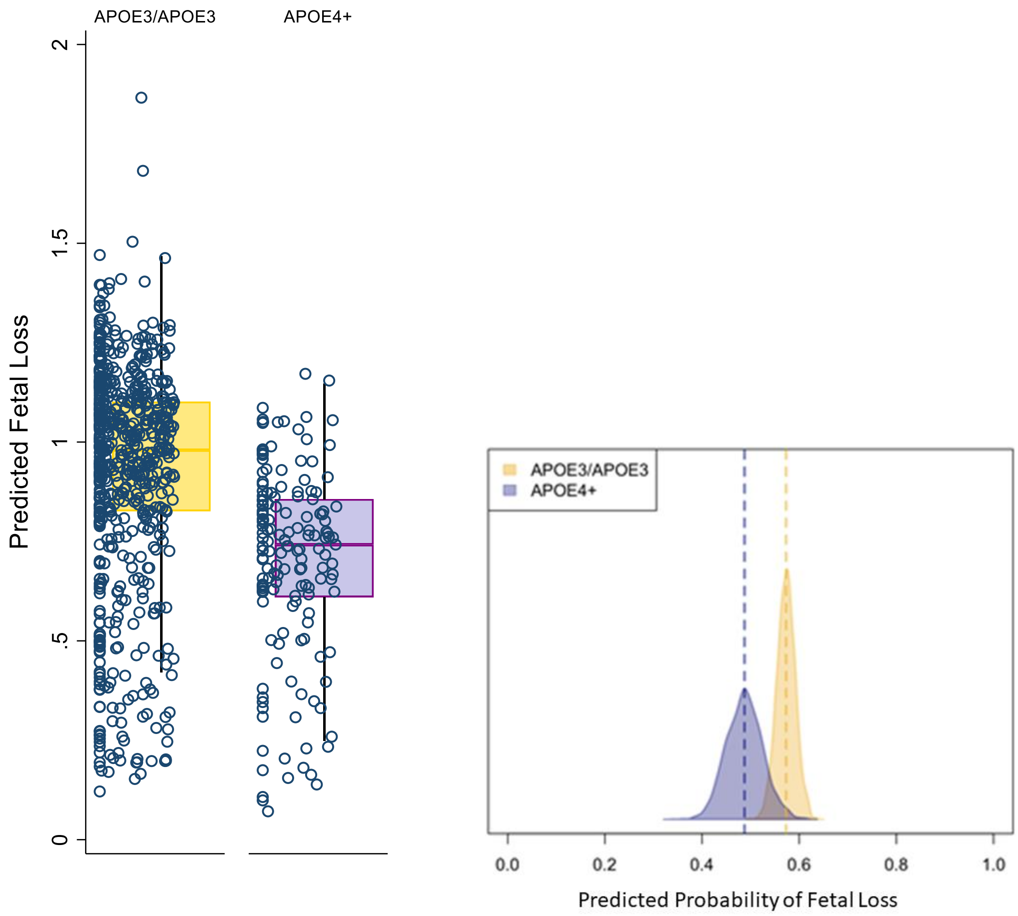

Figure S4: Proportion of *APOE-ε4* alleles by age

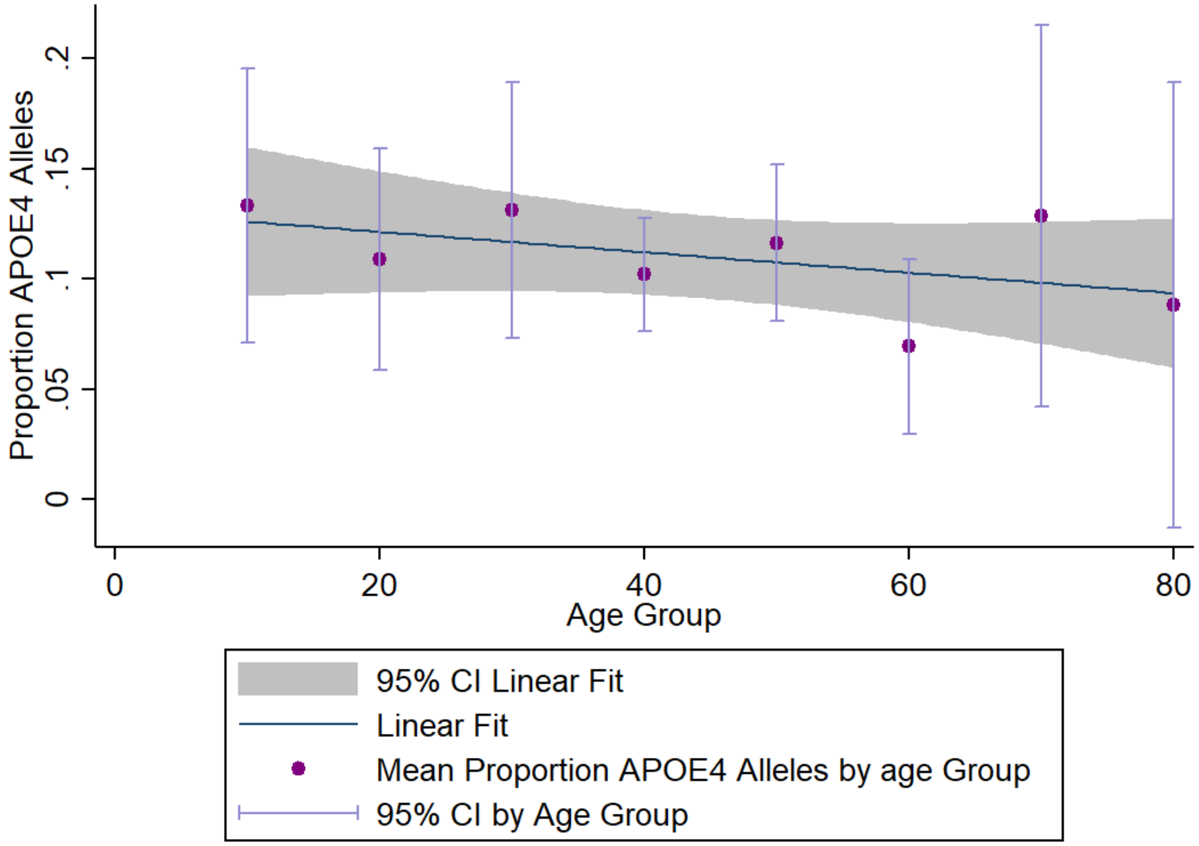

Table S1: Associations between fertility and *APOE-E4* + genotype

	Coefficient	Std Err.	P Value	95%CI	
APOE-ε4+	0.067	0.033	0.033	0.005	0.129
1/Age	-47.375	1.737	<0.001	-50.781	-43.969
BMI	0.007	0.003	0.026	0.001	0.012
Constant	2.023	0.385	<0.001	2.805	3.115

N=795, Pseudo R^2=0.209

Table S2: Associations between fertility and *APOE- ε4* genotypes

	Coefficient	Std Err.	P Value	95%CI	
APOE-ε3/APOE-ε4	0.056	0.033	0.084	-0.007	0.120
APOE-ε4/APOE-ε4	0.188	0.097	0.053	-0.002	0.379
1/Age	-47.386	1.738	<0.001	-50.792	-43.980
BMI	0.007	0.003	0.020	0.001	0.013
Constant	2.953	0.079	<0.001	-0.008	0.120

N=795, Pseudo R^2= 0.2090

Table S3: *APOE* alleles by age and Hardy-Weinberg Equilibrium

	33	34	44	n	p	pq	q	$(p^2)+2pq+(q^2)$
<20	45	14	1	60	0.75	0.233333	0.016667	1.029444
20-30	62	15	1	78	0.794872	0.192308	0.012821	1.016601
30-40	62	15	3	80	0.775	0.1875	0.0375	0.977031
40-50	213	50	2	265	0.803774	0.188679	0.007547	1.023467
50-60	124	33	2	159	0.779874	0.207547	0.012579	1.023456
60-70	89	10	2	101	0.881188	0.09901	0.019802	0.974904
70-80	27	7	1	35	0.771429	0.2	0.028571	0.995918
80+	14	3	0	17	0.823529	0.176471	0	1.031142
Total	636	147	12	795	0.8	0.184906	0.015094	1.010039

APOE-ε4 and Fetal Loss

Research in high-income industrialized settings have not reported associations between fetal loss and *APOE* isoforms (*67, 68*), however, pregnant Tsimane women inhabit a more physically demanding environment than urban women, and have far more pregnancies than US women, and have little or no prenatal medical care. Previous studies reported major differences in immune function during pregnancy for US women versus Tsimane (*69*), and future studies will focus on potential mechanisms related to *APOE-ε4* and immune function during pregnancy and fetal loss.

These data are limited by several factors. First, these are all self reports of fetal loss, and without access to hCG pregnancy tests, the only fetal loss that could be reported would be for women who had missed one or more menses and/or had other pregnancy related symptoms, or who experienced a still birth or heavy bleeding during fetal expulsion. Secondly, we do not have timing on the gestational age at which women experienced fetal loss, and there may be major differences in the cause of fetal loss at 8 weeks versus fetal loss at 30 weeks (*70*). Combined, these limitations make it difficult to assess associations between the *APOE-ε4* allele and fetal loss, and without more fine-grained data it is not possible to test any potential mechanistic role of the *APOE-ε4* allele in fetal loss.

REFERENCES AND NOTES

1. M. S. Beeri, M. Rapp, J. M. Silverman, J. Schmeidler, H. T. Grossman, J. T. Fallon, D. P. Purohit, D. P. Perl, A. Siddiqui, G. Lesser, C. Rosendorff, V. Haroutunian, Coronary artery disease is associated with Alzheimer disease neuropathology in *APOE*4 carriers. *Neurology* **66**, 1399–1404 (2006).

2. G. W. Small, L. M. Ercoli, D. H. S. Silverman, S. C. Huang, S. Komo, S. Y. Bookheimer, H. Lavretsky, K. Miller, P. Siddarth, N. L. Rasgon, J. C. Mazziotta, S. Saxena, H. M. Wu, M. S. Mega, J. L. Cummings, A. M. Saunders, M. A. Pericak-Vance, A. D. Roses, J. R. Barrio, M. E. Phelps, Cerebral metabolic and cognitive decline in persons at genetic risk for Alzheimer's disease. *Proc. Natl. Acad. Sci. U.S.A.* **97**, 6037–6042 (2000).

3. M. Cacciottolo, A. Christensen, A. Moser, J. Liu, C. J. Pike, C. Smith, M. LaDu, P. M. Sullivan, T. E. Morgan, E. Dolzhenko, A. Charidimou, L. O. Wahlund, M. K. Wiberg, S. Shams, G. C. Chiang; Alzheimer's Disease Neuroimaging Initiative, C. E. Finch, The *APOE*4 allele shows opposite sex bias in microbleeds and Alzheimer's disease of humans and mice. *Neurobiol. Aging* **37**, 47–57 (2016).

4. Alzheimer's Association, 2021 Alzheimer's disease facts and figures. *Alzheimers Dement.* **17**, 327–406 (2021).

5. H. N. Yassine, C. E. Finch, *APOE* alleles and diet in brain aging and Alzheimer’s disease. *Front. Aging Neurosci.* **12**, 150 (2020).

6. A. Suchy-Dicey, B. Howard, W. T. Longstreth Jr, E. M. Reiman, D. Buchwald, *APOE* genotype, hippocampus, and cognitive markers of Alzheimer's disease in American Indians: Data from the strong heart study. *Alzheimers Dement.* **18**, 2518–2526 (2022).

7. D. T. A. Eisenberg, C. W. Kuzawa, M. G. Hayes, Worldwide allele frequencies of the human apolipoprotein E gene: Climate, local adaptations, and evolutionary history. *Am. J. Phys. Anthropol.* **143**, 100–111 (2010).

8. M. Fox, ‘Evolutionary medicine’ perspectives on Alzheimer’s disease: Review and new directions. *Ageing Res. Rev.* **47**, 140–148 (2018).

9. D. Iacono, G. C. Feltis, Impact of Apolipoprotein E gene polymorphism during normal and pathological conditions of the brain across the lifespan. *Aging* **11**, 787–816 (2019).

10. S. M. Fullerton, A. G. Clark, K. M. Weiss, D. A. Nickerson, S. L. Taylor, J. H. Stengård, V. Salomaa, E. Vartiainen, M. Perola, E. Boerwinkle, C. F. Sing, Apolipoprotein E variation at the sequence haplotype level: Implications for the origin and maintenance of a major human polymorphism. *Am. J. Human Genet.* **67**, 881–900 (2000).

11. P. B. Medawar, *An Unsolved Problem of Biology* (H. K. Lewis, 1952).

12. B. Charlesworth, *Evolution in Age-Structured Populations* (Cambridge Univ. Press, 1994), vol. 2.

13. G. C. Williams, Pleiotropy, natural selection, and the evolution of senescence. *Evolution* **11**, 398–411 (1957).

14. S. G. Byars, K. Voskarides, Antagonistic pleiotropy in human disease. *J. Mol. Evol.* **88**, 12–25 (2020).

15. R. B. Oriá, P. D. Patrick, J. A. Blackman, A. A. M. Lima, R. L. Guerrant, Role of apolipoprotein E4 in protecting children against early childhood diarrhea outcomes and implications for later development. *Med. Hypotheses* **68**, 1099–1107 (2007).

16. O. G. R. Azevedo, D. T. Bolick, J. K. Roche, R. F. Pinkerton, A. A. M. Lima, M. P. Vitek, C. A. Warren, R. B. Oriá, R. L. Guerrant, Apolipoprotein E plays a key role against cryptosporidial infection in transgenic undernourished mice. *PLOS ONE* **9**, e89562 (2014).

17. B. C. Trumble, J. Stieglitz, A. D. Blackwell, H. Allayee, B. Beheim, C. E. Finch, M. Gurven, H. Kaplan, Apolipoprotein E4 is associated with improved cognitive function in Amazonian forager-horticulturalists with a high parasite burden. *FASEB J.* **31**, 1508–1515 (2017).

18. R. B. Oriá, P. D. Patrick, H. Zhang, B. Lorntz, C. M. de Castro Costa, G. A. C. Brito, L. J. Barrett, A. A. M. Lima, R. L. Guerrant, *APOE*4 protects the cognitive development in children with heavy diarrhea burdens in Northeast Brazil. *Pediatr. Res.* **57**, 310–316 (2005).

19. C. Zhao, K. Strobino, Y. P. Moon, Y. K. Cheung, R. L. Sacco, Y. Stern, M. S.V. Elkind, *APOE* ϵ4 modifies the relationship between infectious burden and poor cognition. *Neurol. Genet.* **6**, e462 (2020).

20. E. van Exel, J. J. E. Koopman, D. . Bodegom, J. J. Meij, P. . Knijff, J. B. Ziem, C. E. Finch, R. G. J. Westendorp, Effect of *APOE* ε4 allele on survival and fertility in an adverse environment. *PLOS ONE* **12**, e0179497 (2017).

21. R. Corbo, L. Ulizzi, R. Scacchi, C. Martinez-Labarga, G. De Stefano, Apolipoprotein E polymorphism and fertility: A study in pre-industrial populations. *Mol. Hum. Reprod.* **10**, 617–620 (2004).

22. R. B. Oriá, J. Z. de Almeida, C. N. Moreira, R. L. Guerrant, J. R. Figueiredo, Apolipoprotein E effects on mammalian ovarian steroidogenesis and human fertility. *Trends Endocrinol. Metab.* **31**, 872–883 (2020).

23. C. F. Sing, J. Davignon, Role of the apolipoprotein E polymorphism in determining normal plasma lipid and lipoprotein variation. *Am. J. Hum. Genet.* **37**, 268–285 (1985).

24. C. E. Finch, T. E. Morgan, Systemic inflammation, infection, *APOE* alleles, and Alzheimer disease: A position paper. *Curr. Alzheimer Res.* **4**, 185–189 (2007).

25. T. Mueller, J. Fischer, R. Gessner, J. Rosendahl, S. Böhm, F. van Bömmel, V. Knop, C. Sarrazin, H. Witt, A. M. Marques, P. Kovacs, D. Schleinitz, M. Stumvoll, M. Blüher, P. Bugert, E. Schott, T. Berg, Apolipoprotein E allele frequencies in chronic and self-limited hepatitis C suggest a protective effect of *APOE* 4 in the course of hepatitis C virus infection. *Liver Int.* **36**, 1267–1274 (2016).

26. A. R. Garcia, C. Finch, M. Gatz, T. Kraft, D. E. Rodriguez, D. Cummings, M. Charifson, K. Buetow, B. A. Beheim, H. Allayee, G. S. Thomas, J. Stieglitz, M. D. Gurven, H. Kaplan, B. C. Trumble, *APOE*4 is associated with elevated blood lipids and lower levels of innate immune biomarkers in a tropical Amerindian subsistence population. *Elife* **10**, e68231 (2021).

27. S. S. Urlacher, P. T. Ellison, L. S. Sugiyama, H. Pontzer, G. Eick, M. A. Liebert, T. J. Cepon-Robins, T. E. Gildner, J. J. Snodgrass, Tradeoffs between immune function and childhood growth among Amazonian forager-horticulturalists. *Proc. Natl. Acad. Sci. U.S.A.* **115**, E3914–E3921 (2018).

28. R. B. Oriá, P. D. Patrick, M. O. B. Oriá, B. Lorntz, M. R. Thompson, O. G. R. Azevedo, R. N. B. Lobo, R. F. Pinkerton, R. L. Guerrant, A. A. M. Lima, *APOE* polymorphisms and diarrheal outcomes in Brazilian shanty town children. *Braz. J. Med. Biol. Res.* **43**, 249–256 (2010).

29. J. Bongaarts, A framework for analyzing the proximate determinants of fertility. *Popul. Dev. Rev.* **4**, 105–132 (1978).

30. J. W. Wood, *Dynamics of Human Reproduction: Biology, Biometry, Demography*. (Routledge, 1994).

31. C. Valeggia, P. T. Ellison, Interactions between metabolic and reproductive functions in the resumption of postpartum fecundity. *Am. J. Hum. Biol.* **21**, 559–566 (2009).

32. R. M. Corbo, R. Scacchi, M. Cresta, Differential reproductive efficiency associated with common apolipoprotein E alleles in postreproductive-aged subjects. *Fertil. Steril.* **81**, 104–107 (2004).

33. L. U. Gerdes, C. Gerdes, P. S. Hansen, I. C. Klausen, O. Færgeman, Are men carrying the apolipoprotein ε4-or ε2 allele less fertile than ε3ε3 genotypes? *Hum. Genet.* **98**, 239–242 (1996).

34. G. Jasienska, P. T. Ellison, A. Galbarczyk, M. Jasienski, M. Kalemba-Drozdz, M. Kapiszewska, I. Nenko, I. Thune, A. Ziomkiewicz, Apolipoprotein E (*APOE*) polymorphism is related to differences in potential fertility in women: A case of antagonistic pleiotropy? *Proc. R. Soc. B Biol. Sci.* **282**, 20142395 (2015).

35. S. Vasunilashorn, C. E. Finch, E. M. Crimmins, S. A. Vikman, J. Stieglitz, M. Gurven, H. Kaplan, H. Allayee, Inflammatory gene variants in the Tsimane, an indigenous Bolivian population with a high infectious load. *Biodemography Soc. Biol.* **57**, 33–52 (2011).

36. M. Gurven, Infant and fetal mortality among a high fertility and mortality population in the Bolivian Amazon. *Soc. Sci. Med.* **75**, 2493–2502 (2012).

37. M. A. Martin, G. Garcia, H. S. Kaplan, M. D. Gurven, Conflict or congruence? Maternal and infant-centric factors associated with shorter exclusive breastfeeding durations among the Tsimane. *Soc. Sci. Med.* **170**, 9–17 (2016).

38. J. Heeren, T. Grewal, A. Laatsch, N. Becker, F. Rinninger, K.A. Rye, U. Beisiegel, Impaired recycling of apolipoprotein E4 is associated with intracellular cholesterol accumulation. *J. Biol. Chem.* **279**, 55483–55492 (2004).

39. Y. Kesäniemi, C. Ehnholm, T. A. Miettinen, Intestinal cholesterol absorption efficiency in man is related to apoprotein E phenotype. *J. Clin. Invest.* **80**, 578–581 (1987).

40. H. Kaplan, R. C. Thompson, B. C. Trumble, L. S. Wann, A. H. Allam, B. Beheim, B. Frohlich, M. L. Sutherland, J. D. Sutherland, J. Stieglitz, D. E. Rodriguez, D. E. Michalik, C. J. Rowan, G. P. Lombardi, R. Bedi, A. R. Garcia, J. K. Min, J. Narula, C. E. Finch, M. Gurven, G. S. Thomas, Coronary atherosclerosis in indigenous South American Tsimane: A cross-sectional cohort study. *Lancet* **389**, 1730–1739 (2017).

41. M. Gatz, W. J. Mack, H. C. Chui, E. M. Law, G. Barisano, M. L. Sutherland, J. D. Sutherland, D. E. Rodriguez, R. Q. Gutierrez, J. C. Adrian, J. B. Cuata, A. R. Borenstein, E. E. Walters, A. Irimia, C. J. Rowan, L. S. Wann, A. H. Allam, R. C. Thompson, M. I. Miyamoto, D. E. Michalik, D. K. Cummings, E. Seabright, A. R. Garcia, P. L. Hooper, T. S. Kraft, C. E. Finch, G. S. Thomas, J. Stieglitz, B. C. Trumble, M. D. Gurven, H. Kaplan, Prevalence of dementia and mild cognitive impairment in indigenous Bolivian forager-horticulturalists. *Alzheimer's Dement.* **19**, 44–55 (2023).

42. R. D. Moir, R. Lathe, R. E. Tanzi, The antimicrobial protection hypothesis of Alzheimer's disease. *Alzheimers Dement.* **14**, 1602–1614 (2018).

43. G. Jasienska, R. G. Bribiescas, A.-S. Furberg, S. Helle, A. Núñez-de la Mora, Human reproduction and health: An evolutionary perspective. *Lancet* **390**, 510–520 (2017).

44. M. Van Haute, E. Rondilla II, J. L. Vitug, K. D. Batin, R. E. Abrugar, F. Quitoriano, K. D. Merced, T. Maaño, J. Higa, J. G. Almoro, D. Ternida, J. T. Cabrera, Assessment of a proposed BMI formula in predicting body fat percentage among Filipino young adults. *Sci. Rep.* **10**, 21988 (2020).

45. T. S. Kraft, J. Stieglitz, B. C. Trumble, M. Martin, H. Kaplan, M. Gurven, Nutrition transition in 2 lowland Bolivian subsistence populations. *Am. J. Clin. Nutr.* **108**, 1183–1195 (2018).

46. K. A O’Connor, R. Ferrell, E. Brindle, B. Trumble, J. Shofer, D. J. Holman, M. Weinstein, Progesterone and ovulation across stages of the transition to menopause. *Menopause* **16**, 1178–1187 (2009).

47. M. D. Gurven, R. J. Davison, Periodic catastrophes over human evolutionary history are necessary to explain the forager population paradox. *Proc. Natl. Acad. Sci. U.S.A.* **116**, 12758–12766 (2019).

48. B. C. Trumble, C. E. Finch, The exposome in human evolution: From dust to diesel. *Q. Rev. Biol.* **94**, 333–394 (2019).

49. A. D. Blackwell, B. C. Trumble, I. M. Suarez, J. Stieglitz, B. Beheim, J Josh Snodgrass, H. Kaplan, M. Gurven, Immune function in Amazonian horticulturalists. *Ann. Hum. Biol.* **43**, 382–396 (2016).

50. A. J. Lea, A. Garcia, J. Arevalo, J. F. Ayroles, K. Buetow, S. W. Cole, D. Eid Rodriguez, M. Gutierrez, H. M. Highland, P. L. Hooper, A. Justice, T. Kraft, K. E. North, J. Stieglitz, H. Kaplan, B. C. Trumble, M. D. Gurven, Natural selection of immune and metabolic genes associated with health in two lowland Bolivian populations. *Proc. Natl. Acad. Sci. U.S.A.* **120**, e2207544120 (2023).

51. H. Mostafavi, T. Berisa, F. R. Day, J. R. B. Perry, M. Przeworski, J. K. Pickrell, Identifying genetic variants that affect viability in large cohorts. *PLOS Biol.* **15**, e2002458 (2017).

52. M. Kacperczyk, A. Kmieciak, E. M. Kratz, The role of *APOE* expression and variability of its glycosylation in human reproductive health in the light of current information. *Int. J. Mol. Sci.* **22**, 7197 (2021).

53. P. L. Hooper, M. Gurven, J. Winking, H. S. Kaplan, Inclusive fitness and differential productivity across the life course determine intergenerational transfers in a small-scale human society. *Proc. R. Soc. B Biol. Sci.* **282**, 20142808 (2015).

54. R. Davison, M. Gurven, The importance of elders: Extending Hamilton’s force of selection to include intergenerational transfers. *Proc. Natl. Acad. Sci. U.S.A.* **119**, e2200073119 (2022).

55. M. Shinohara, T. Kanekiyo, M. Tachibana, A. Kurti, M. Shinohara, Y. Fu, J. Zhao, X. Han, P. M. Sullivan, G. W. Rebeck, J. D. Fryer, M. G. Heckman, G. Bu, *APOE*2 is associated with longevity independent of Alzheimer's disease. *eLife* **9**, e62199 (2020).

56. M. D. Gurven, D. E. Lieberman, WEIRD bodies: Mismatch, medicine and missing diversity. *Evol. Hum. Behav.* **41**, 330–340 (2020).

57. C. R. A. Mondadori, D. J.-F. de Quervain, A. Buchmann, H. Mustovic, M. A. Wollmer, C. F. Schmidt, P. Boesiger, C. Hock, R. M. Nitsch, A. Papassotiropoulos, K. Henke, Better memory and neural efficiency in young apolipoprotein E ε4 carriers. *Cereb. Cortex* **17**, 1934–1947 (2007).

58. G. H. Weissberger, D. A. Nation, C. P. Nguyen, M. W. Bondi, S. D. Han, Meta-analysis of cognitive ability differences by apolipoprotein e genotype in young humans. *Neurosci. Biobehav. Rev.* **94**, 49–58 (2018).

59. K. R. Smith, H. A. Hanson, G. P. Mineau, S. S. Buys, Effects of BRCA1 and BRCA2 mutations on female fertility. *Proc. R. Soc. B Biol. Sci.* **279**, 1389–1395 (2012).

60. M. Gurven, J. Stieglitz, B. Trumble, A. D. Blackwell, B. Beheim, H. Davis, P. Hooper, H. Kaplan, The Tsimane health and life history project: Integrating anthropology and biomedicine. *Evol. Anthropol.* **26**, 54–73 (2017).

61. M. Gurven, M. Costa, Ben Trumble, J. Stieglitz, B. Beheim, D. Eid Rodriguez, P. L. Hooper, H. Kaplan, Health costs of reproduction are minimal despite high fertility, mortality and subsistence lifestyle. *Sci. Rep.* **6**, 30056 (2016).

62. M. E. Costa, B. Trumble, H. Kaplan, M. D. Gurven, Child nutritional status among births exceeding ideal family size in a high fertility population. *Matern. Child Nutr.* **14**, e12625 (2018).

63. A. D. Blackwell, M. A. Tamayo, B. Beheim, B. C. Trumble, J. Stieglitz, P. L. Hooper, M. Martin, H. Kaplan, M. Gurven, Helminth infection, fecundity, and age of first pregnancy in women. *Science* **350**, 970–972 (2015).

64. M. Gurven, H. Kaplan, A. Z. Supa, Mortality experience of Tsimane Amerindians of Bolivia: Regional variation and temporal trends. *Am. J. Hum. Biol.* **19**, 376–398 (2007).

65. K. Hill, A. M. Hurtado, *Ache Life History: The Ecology and Demography of a Foraging People* (Routledge, 2017).

66. N. Howell, *Demography of the Dobe! Kung* (Routledge, 2017).

67. S. Bianca, B. Barrano, N. Cutuli, L. Indaco, A. Cataliotti, G. Milana, C. Barone, G. Ettore, No association between apolipoprotein E polymorphisms and recurrent pregnancy loss. *Fertil. Steril.* **93**, 276 (2010).

68. J. Rynekrova, D. Kasparova, V. Adamkova, T. Fait, J. A. Hubacek, Analysis of the potential role of Apolipoprotein E polymorphism in genetic predisposition to spontaneous abortion. *Am. J. Reprod. Immunol.* **67**, 179–183 (2012).

69. C. Hové, B. C. Trumble, A. S. Anderson, J. Stieglitz, H. Kaplan, M. D. Gurven, A. D. Blackwell, Immune function during pregnancy varies between ecologically distinct populations. *Evol. Med. Public Health* **2020**, 114–128 (2020).

70. D. J. Holman, *Total Fecundability and Fetal Loss in Rural Bangladesh* (The Pennsylvania State University, 1996).
